# Design and In Vitro Activity of Furcellaran/Chitosan Multilayer Microcapsules for the Delivery of Glutathione and Empty Model Multilayer Microcapsules Based on Polysaccharides

**DOI:** 10.3390/ma17092047

**Published:** 2024-04-26

**Authors:** Mariola Drozdowska, Ewelina Piasna-Słupecka, Aleksandra Such, Kinga Dziadek, Paweł Krzyściak, Tomasz Kruk, Dorota Duraczyńska, Małgorzata Morawska-Tota, Ewelina Jamróz

**Affiliations:** 1 Department of Human Nutrition and Dietetics, Faculty of Food Technology, University of Agriculture, Balicka 122, 30-149 Kraków, Poland; aleksandra.such@urk.edu.pl (A.S.); kinga.dziadek@urk.edu.pl (K.D.); 2 Department of Mycology, Collegium Medicum, Jagiellonian University, Czysta 18, 31-121 Kraków, Poland; pawel.krzysciak@uj.edu.pl; 3 Jerzy Haber Institute of Catalysis and Surface Chemistry, Polish Academy of Sciences, Niezapominajek 8, 30-239 Kraków, Poland; tomasz.kruk@ikifp.edu.pl (T.K.); dorota.duraczynska@ikifp.edu.pl (D.D.); 4 Department of Sports Medicine & Human Nutrition, Faculty of Physical Education and Sport, University of Physical Education, Jana Pawła II 78, 31-571 Kraków, Poland; malgorzata.morawska@awf.krakow.pl; 5 Department of Chemistry, University of Agriculture, Balicka 122, 30-149 Kraków, Poland; ewelina.jamroz@urk.edu.pl; 6 Department of Product Packaging, Cracow University of Economics, Rakowicka 27, 31-510 Kraków, Poland

**Keywords:** encapsulation, glutathione, furcellaran/chitosan microcapsules, multilayer capsules, cytotoxicity, apoptosis

## Abstract

In this study, multilayer microcapsules (two-layer and four-layer) based on furcellaran (FUR) and chitosan (CHIT) were produced, enclosing a tripeptide with an antioxidant effect—glutathione—in different concentrations. In addition, for the first time, an empty, four-layer microcapsule based on CHIT and FUR (ECAPS) was obtained, which can be used to contain sensitive, active substances of a hydrophobic nature. Layering was monitored using zeta potential, and the presence of the resulting capsules was confirmed by SEM imaging. In the current study, we also investigated whether the studied capsules had any effect on the Hep G2 cancer cell line. An attempt was also made to identify the possible molecular mechanism(s) by which the examined capsules suppressed the growth of Hep G2 cells. In this report, we demonstrate that the capsules suppressed the growth of cancer cells. This mechanism was linked to the modulation of the AKT/PI3K signaling pathway and the induction of the G2/M arrest cell cycle. Furthermore, the results indicate that the tested multilayer microcapsules induced cell death through an apoptotic pathway.

## 1. Introduction

World interest in biopolymers has increased in recent years, not only due to the desire to replace petrochemical-based polymeric materials, but also as a result of innovative developments in various biomedical, environmental, and economic fields. Naturally derived polymers possess numerous advantageous attributes, including their ability to degrade naturally, their minimal harmful effects on living organisms, their compatibility with biological systems, and their potential for reuse, making them preferable candidates for producing a diverse range of coatings (e.g., films and nano- or microcapsules) over traditional fossil-based materials used in membrane products [1].

Polysaccharide-based nano- or microcapsules are a promising alternative for the safe and controlled administration of active substances. Polysaccharide capsules are characterized by no toxicity, low density, high load capacity, and high colloidal stability [2]. Currently, various approaches are being used to produce polysaccharide-based nanocapsules, including the most commonly used layer-by-layer (LbL) method [3,4], emulsion polymerization [5], interfacial reaction [6], and matrix polymerization [7]. The LbL method is a convenient tool for producing nanoparticles targeted at the delivery of anticancer drugs (nanocapsules). It relies on the sequential adsorption of oppositely charged polyelectrolytes. The number of layers can be readily adjusted by controlling the pH and the salt concentration, which also influence the size of the nano- or microcapsules and the release time of the active substance [1].

Chitosan (CHIT) is a biodegradable polymer derived from the exoskeletons of crustaceans [8]. Chitosan (CHIT) exhibits poor solubility in water, alkaline solutions, and organic solvents owing to the establishment of intermolecular hydrogen bonding among its molecules. However, it exhibits solubility in dilute acidic solutions, which is mainly due to the protonation of its amino groups in an aqueous acidic environment. [9].

In addition, both laboratory based (in vitro) and live organism (in vivo) investigations have confirmed the antibacterial efficacy of chitosan against various microorganisms, encompassing bacteria, algae, fungi, and yeasts [10].

Furcellaran (FUR) is a sulfate polysaccharide with a negative charge that is extracted from the red algae *Furcellaria lumbricalis*. FUR was approved by the European Commission (Commission Regulation (EU) No. 231/2012) as a food additive and given the number E 407. This polysaccharide easily interacts with starch, gelatin, chitosan, and other compounds, resulting in the formation of functional biopolymer films [11]. FUR is soluble in most aqueous conditions, including those present in the stomach and small intestine.

The solubility of these two compounds limits their use in the delivery of active substances. The electrostatic interaction between the oppositely charged CHIT and FUR layers prompts their assembly, resulting in the creation of spherical multilayer nanocapsules [12]. Such capsules are distinguished by the formation of robust intermolecular bonds between the functional groups of biopolymers, which impede the rapid dissolution of the nanocapsules or the release of the active substance. Therefore, FUR and CHIT present significant potential for encapsulating active compounds and producing nanocapsules of controlled sizes, achieved by a simple adjustment of the deposited layer count [1,13].

The obtained research results indicate many possible applications of modern structures as platforms for active substances. From the above mentioned biosubstrates, we obtained layers of capsules in which glutathione was enclosed. Glutathione (GSH), a tripeptide found abundantly across the spectrum of living organisms, plays pivotal roles in numerous essential cellular functions. It is integral to drug and endogenous substance metabolism and preserving the structural integrity of erythrocytes [14]. In addition, glutathione is an essential component of antioxidant defense systems and participates in eradicating harmful substances. Glutathione deficiency has been found in people with chronic fatigue syndrome, cancer, Alzheimer’s disease, Parkinson’s disease, chronic infections, and liver diseases. Moreover, athletes develop glutathione deficiencies that need to be supplemented from the outside. Research results indicate that taken orally, glutathione is degraded very quickly and loses its beneficial properties [15]. Enclosing glutathione in a biopolymer colloidal system will protect it from the action of digestive enzymes, allowing it to reach the intestines in an unchanged form. A glutathione delivery system designed in this way will provide protection against environmental factors and controlled release of this tripeptide.

The primary objective of this study was to develop two different types of capsules that could potentially be used to encapsulate sensitive hydrophilic and hydrophobic active ingredients. For the first time, glutathione was enclosed in a biopolymer capsule. Also for the first time, an empty capsule with FUR/CHIT layers was designed. The capsule is designed to protect the sensitive ingredient glutathione against the effects of the gastrointestinal environment. However, the empty capsule must be safe and non-toxic in order to contain other sensitive ingredients in further stages. The first type of capsule, which contained the active hydrophilic substance glutathione in its core, consisted of two or four biopolymer layers (FUR/CHIT)_n_. The second type of capsule was based on liquid core encapsulation using multilayer polysaccharide adsorption (CHIT/FUR)_n_. This was a model capsule for encapsulating hydrophobic substances. The next goal was to evaluate the cytotoxicity of digested and absorbed glutathione in examined concentrations (GSH5, GSH25) and glutathione enriched (FUR/CHIT)_n_ capsules with two and four layers (2L5, 2L25, 4L25) and model empty microcapsules (ECAPS) on normal and cancer cell lines in vitro. As far as we know, this study is the first to evaluate the cytotoxicity of glutathione enriched (FUR/CHIT)_n_ and empty capsules (CHIT/FUR)_n_ and their potential anticancer activity.

## 2. Materials and Methods

### 2.1. Materials for Preparing Capsules

L-Glutathione reduced (GSH) (CAS number 70-18-8) was supplied by Sigma Aldrich (St. Louis, MO, USA). Furcellaran (FUR) (type 7000) was procured from Est-Agar AS (Karla village, Estonia). The chemical content composition of FUR (Mw= 2.951 × 105) was: carbohydrates 79.61%; protein 1.18% and fat 0.24%. Chitosan (CHIT) (molecular weight ~890,000, CAS number 9012-76-4 ≥ 90% deacetylation, 100–300 cP viscosity; particle size ≤ 100 mesh) was supplied by POL-AURA (Zabrze, Poland). Sodium hydroxide (>99%) was purchased from Sigma Aldrich (St. Louis, MO, USA).

### 2.2. Preparation of Biopolymer Solution

FUR and CHIT solutions were prepared at 500 ppm in 0.005 M NaCl; the FUR was dissolved in distilled water and the chitosan in 2% acetic acid. The solutions prepared in this way were subjected to ultrasound in order to better dissolve the solutions. Glutathione was prepared in two concentrations of 5 mg/mL H_2_O and 25 mg/mL H_2_O in 0.005 M NaCl.

### 2.3. Preparation of Capsules with Different Number of Layers Enclosed with Glutathione

Several capsule options were prepared using the layer-by-layer method (LbL). The first option was a two-layered capsule with varying glutathione contents of either 5 mg/mL H_2_O or 25 mg/mL H_2_O. The glutathione solutions prepared in this manner constituted the core of the capsules, on which the first layer of FUR was applied, and then the layer of CHIT, while mixing with a magnetic stirrer at 400 rpm. The capsules prepared in this way were named 2L5 and 2L25, respectively. GSH encapsulation was achieved by layering biopolymers (GSH/FUR/CHIT) at a ratio of 0.3/0.08/0.08. Another type of capsules was prepared by layering a glutathione core of 25 mg/mL first with a layer of negatively charged FUR, then positively charged CHIT, then FUR, and a final and fourth layer made of CHIT. These capsules were named 4L25. GSH encapsulation was achieved by layering biopolymers (GSH/FUR/CHIT/FUR/CHIT) at a ratio of 0.3/0.08/0.08/0.25/0.26. The preparation of the capsules was monitored using Malvern Zetasizer equipment, controlling the zeta potential.

### 2.4. Preparation of Empty Capsules with Four Layers

Another approach to creating stable capsules was based on the encapsulation of a liquid core by alternating deposition layers of oppositely charged polysaccharides. Using the saturation method, (CHIT/FUR)_n_ microcapsules were formed on a liquid core. The liquid core was AOT (AOT—docusate sodium salt) in chloroform. Hydrophobic active ingredients can potentially be introduced into capsules produced using this method. The capsules were formed using a modified method demonstrated by Szczepanowicz et al. [16]. The oil phase formation was created by diluting 180 g/dm^3^ AOT in CHCl_3_ (chloroform). Due to its toxicity, the solvent (CHCl_3_) was removed by evaporation from dispersions of capsules after the formation of the emulsion. The polysaccharides were dissolved in 0.005 M NaCl to receive a polysaccharide solution with a concentration of 0.5 g/dm^3^ without changing the pH value. The capsules were obtained by adding the AOT/CHCl_3_ to the CHIT solution during mixing (rotation speed 400 rpm). The best ratio of AOT to CHIT was set by estimating the zeta potential of the emulsion drops and determining their stability. The stable emulsions were formed when the zeta potential of the drops with a deposited polysaccharide shell was very close to the zeta potential of the same polysaccharide in the solution. Then the quantity of free polysaccharides was minimized. It is very important to note that in this method of formulating emulsion cores, the core escapes the presence of surfactants, which are usually toxic to cells. After deposition of the first polysaccharide layer, the subsequent layers of polysaccharide were created via the LbL method, using saturation [17]. The polysaccharide shells of the capsules were formed by subsequent deposition of polysaccharides from their solutions (without a rinsing step). The volumes of the polysaccharide solution which were used to prepare every layer were obtained empirically by analyzing the results of the zeta potential measurements. The ideal volume was reached when the zeta potential closely matched that of the polysaccharide in the solution.

Abbreviations used in the publication:

- FUR/CHIT two-layer capsule enriched in GSH (5 mg or 25 mg/mL)—2L5 and 2L25
- (FUR/CHIT)_2_ four-layer capsule enriched in GSH (5 mg or 25 mg/mL)—4L5 and 4L25
- (CHIT/FUR)_2_ empty capsule—ECAPS

**Figure 1:**
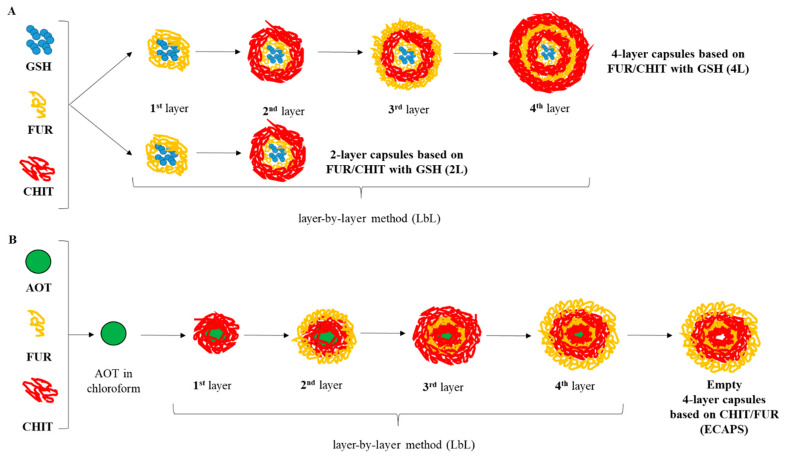
Procedure for obtaining (**A**) two- and four-layer (FUR/CHIT)_n_ capsules with closed glutathione and (**B**) empty model capsules with (CHIT/FUR)_n_ layers.

The scheme of obtaining different types of capsules is shown in Figure 1.

### 2.5. Particle Size and Zeta Potential

Characterization of the polysaccharide solutions and microcapsules was carried out using dynamic light scattering (DLS) (Zetasizer Ultra Red, Malvern Instruments Ltd., Worcestershire, UK).

### 2.6. SEM

Scanning electron microscopy (SEM) analysis was conducted with the aid of a JEOL JSM-7500F instrument (JEOL, Tokyo, Japan). SEM images of the specimens coated with 20 nm of Cr were recorded.

### 2.7. Simulated In Vitro Digestion Model of Gastrointestinal Tract

The material underwent a two-step in vitro digestion process, following the protocol outlined by Minekus et al. [18]. Finally, intestinal microflora was introduced, followed by incubation at 37 °C for 16 h.

### 2.8. Simulated In Vitro Absorption Model of Gastrointestinal Tract

The in vitro digested product underwent sterilization via filtration using nitrocellulose filters. The absorption of capsules was performed using the Caco-2 human colon cancer cell line, as previously reported [19]. Caco-2 monolayers with a trans-epithelial electrical resistance (TEER) exceeding 200 Ω/cm^2^ were employed for permeability assessments. The digested material was applied to the upper surface of the Caco-2 cell monolayer for a duration of 2 h. Subsequently, the filtrate was gathered for analysis.

### 2.9. Cell Culture

This research was conducted using the BJ human normal cell line (CRL-2522™), the Hep G2 human liver cancer cell line (HB-8065™), and the Caco-2 human colon cancer cell line (HTB-37™). Cell lines were purchased from the American Type Culture Collections (ATCC, Manassas, VA, USA). Cells were cultured under controlled conditions in an incubator (NuAire, Plymouth, MN, USA) and in an appropriate medium as per the ATCC protocol.

### 2.10. Cell Treatment

For cytotoxicity and cell proliferation evaluations, the BJ and Hep G2 cell lines were seeded onto 96-well plates at a density of 8 × 10^4^ cells per well. After 24 h of incubation, the growth medium was replaced with filtrates obtained from digestion and absorption: GSH5 5 mg/mL glutathione; GSH25 25 mg/mL glutathione; 2L5 5 mg/mL glutathione enriched FUR/CHIT capsules with two layers; 2L25 25 mg/mL glutathione enriched (FUR/CHIT) capsules with two layers; 4L25 25 mg/mL glutathione enriched (FUR/CHIT)_2_ capsules with four layers; and ECAPS empty (CHIT/FUR)_2_ capsules, for 24 and 48 h of incubation. For use in a Muse^®^ flow cytometer, the Hep G2 cell line was seeded onto six-well plates at a density of 2 × 10^5^ cells per well. The growth medium was replaced with filtrates (digested and absorbed capsules) 24 h after cell seeding, after which the cells were given 48 h of incubation. Cells grown in complete culture medium without any capsules, referred to as untreated cells (UC), served as the negative control for all experiments. A 2% solution of Triton X-100 (Sigma Aldrich, St. Louis, MO, USA) served as the positive control, while staurosporine [1.5 µM] (Sigma Aldrich, St. Louis, MO, USA) was used to induce apoptosis and served as a positive control for this process.

### 2.11. Cell Proliferation Assessment

Cell proliferation was assessed using a commercially available colorimetric and immunoassay BrdU Kit (Sigma Aldrich, St. Louis, MO, USA), following the instructions provided by the manufacturer. Proliferation analysis was conducted using a spectrophotometer.

### 2.12. Cytotoxicity Assay

The capsules’ cytotoxicity was evaluated using the Cytotoxicity Detection Kit (Lactate dehydrogenase, LDH) from Roche (Basel, Switzerland), following the manufacturer’s procedure.

### 2.13. Muse Flow Cytometer Analysis

The cells were labeled as per the manufacturer’s instructions for the following analyses: Muse^®^ PI3K/MAPK Dual Pathway Activation Kit, Muse^®^ Annexin V & Dead Cell Assay Kit, Muse^®^ Caspase-3/7 Kit, and Muse^®^ Cell Cycle Kit. Subsequent analyses were conducted using a Muse^®^ Cell Analyzer (Merck, Kenilworth, NJ, USA).

### 2.14. Statistical Analysis

The experiments were conducted across a minimum of three independent trials, with each measurement taken in triplicate. The normality of distribution was assessed using the Shapiro–Wilk test. Based on the results, a one-way analysis of variance (ANOVA) was employed. Duncan’s post hoc test was used to determine differences, with significance set at *p* ≤ 0.05. Statistical analysis was carried out using Statistica v.13.3 software (Stat-Soft, Tulsa, OK, USA).

## 3. Results and Discussion

### 3.1. Preparation of Capsules

**Figure 2:**
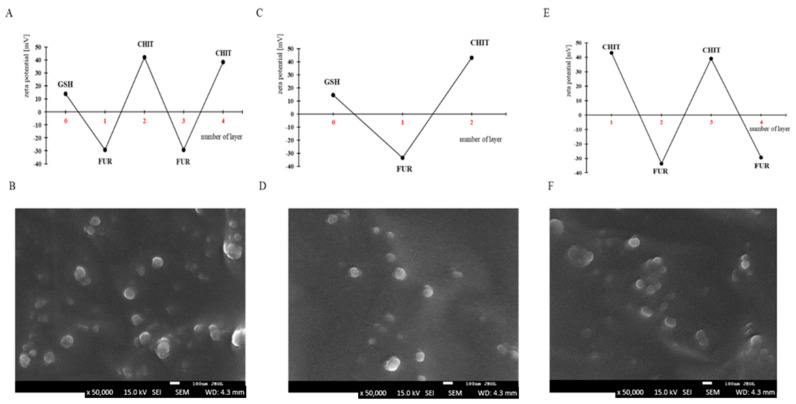
(**A**) ζ-Potential change and; (**B**) SEM photos of capsules (4L25); (**C**) ζ-Potential change and; (**D**) SEM photos of capsules (2L25), (**E**) ζ-Potential change and; (**F**) SEM photos of model empty capsules (ECAPS).

An attempt was made to prepare capsules based on furcellaran (500 ppm in 0.005 M NaCl; size 153.8 nm; PI-0.3071) and chitosan (500 ppm in 0.005 M NaCl; size 230 nm; PI 0.3391), in which glutathione (5 mg or 25 mg/mL in 0.005 M NaCl; 107.4–190.0 nm; PI-0.2244–0.4531), a tripeptide with high antioxidant potential, was enclosed. In previous studies, furcellaran and chitosan were used as opposing biopolymers to obtain nanocapsules with the cancer drug doxorubicin encapsulated inside them. The targeted delivery of doxorubicin was achieved by modifying the surface of the nanocapsules with a homing peptide (seq. SMSIARLC) [1]. Both biopolymers have very good properties for the production of capsule shells. In this work, to encapsulate glutathione in two and four layers of biopolymers, the LbL method was used. In addition, an empty capsule based on furcellaran and chitosan was developed to serve as a model carrier for enclosing sensitive hydrophobic active ingredients. The layering of the two- and four-layer capsules was monitored by measuring the zeta potential of each biopolymer (Figure 2A,C). Glutathione has a positive charge, and therefore the initial stage of capsule formation consisted of the adsorption of a negatively charged furcellaran solution. The next step was to apply a positively charged chitosan solution. Thus, the zeta potential changed between negative (less than −30 mV) and positive (more than +30 mV) values after the deposition of furcellaran and chitosan, respectively. In this way, glutathione was enclosed in two- and four-layer capsules. The zeta potential values provide information about the stability of the obtained solutions. If the zeta potential values are greater than +30 mV and less than −30 mV, they are considered to be generally stable, indicating strong repulsive forces that prevent aggregation from occurring in the solution [20]. In the presented work, the obtained zeta potential results indicate a stable suspension of capsules due to the presence of strong electrostatic repulsions. Moreover, the layering did not significantly reduce the zeta potential value, suggesting that aggregation did not occur. The method used in this work allows the active ingredients to be protected against various external factors. Li et al. [21] enclosed a *Perinereis aibuhitensis* extract (PaE) in alginate hydrogel/Arabic gum/gelatin-based composite capsules for the protection of sensitive ingredients.

Confirmation of targeted capsule formation was conducted through SEM analysis, with representative SEM images compiled in Figure 2. Across all cases, regardless of the synthesis method, the samples exhibited a consistent morphology. Polysaccharide capsules were observed as spherical structures with smooth surfaces and well-defined boundaries. Moreover, they displayed uniformity in size, typically ranging from 80 to 100 nm. However, their distribution was not entirely uniform, occasionally forming aggregates of particles.

The procedure of polysaccharide adsorption on emulsion droplets required the special selection of a surfactant that has good properties as an emulsifier and ensures the use of the surface charge for the sequential adsorption of polysaccharides without losing the stability of the emulsion. AOT, a Food and Drug Administration-approved oil-soluble surfactant with a negative charge, was adopted as the emulsifier to produce oil emulsion droplets (capsule cores). These droplets were stabilized by AOT/polysaccharide interfacial complexes. For the formation of AOT model empty microcapsules in chloroform (180 g/dm^3^), a solution was added to 6 mL of the aqueous CHIT solution (0.5 g/dm^3^) during continuous mixing (magnetic stirrer at 400 rpm). Chloroform was evaporated from the capsule suspensions post-formation. The average drop size estimated using DLS was approx. 90–130 nm with a polydispersity index (PDI) of <0.4. The zeta potential of the emulsion drops was 43.2 mV. The preparation of the multilayer polysaccharide shells on these AOT/CHIT stabilized capsules was done by subsequent adsorption of polysaccharides from their solutions without the indirect rinsing step, as detailed in the procedure above. In Figure 2E, a typical zigzag dependence is shown, relating the zeta potential of capsules to the deposition of subsequent CHIT and FUR layers. The presented layer-to-layer variations of zeta potential were from c.a. 30–40 mV for the polycation to c.a. −30–−40 mV for the polyanion layers. They give evidence of the creation of consecutive layers of the capsule shells. The average size of capsules with four (CHIT/FUR)_2_ layers, which was measured via the DLS method, was about 370 nm (PDI: 0.402). An example of an SEM micrograph of the same (CHIT/FUR)_2_ capsules is presented in Figure 2F. The average stability of the expected capsules is up to about 10 days. In that time, no significant changes were observed in the sizes and zeta potentials measured by the DLS technique. The amounts of chloroform used were negligible; their effects on cytotoxicity were insignificant and undetectable, and in any case they evaporated. In the produced capsules, various types of hydrophobic compounds/drugs can be included, e.g., antimicrobial or anticancer agents, which could be very interesting to the drug delivery industry.

### 3.2. Impact on Cell Proliferation

**Figure 3:**
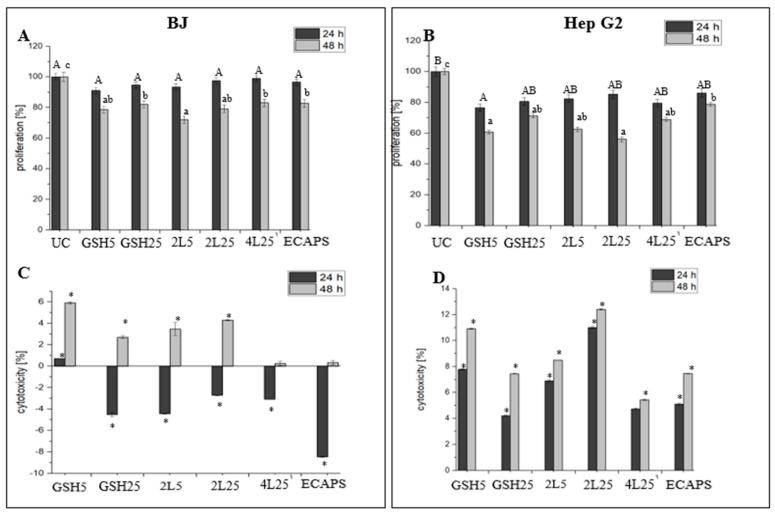
Effect of analyzed capsules on BJ normal cells (**A**) and Hep G2 cancer cells (**B**) proliferation and cytotoxicity of studied capsules against BJ normal cells (**C**) and Hep G2 cancer cell line (**D**). UC, untreated control. GSH5, 5 mg/mL glutathione. GSH25, 25 mg/mL glutathione. 2L5, 5 mg/mL glutathione enriched FUR/CHIT capsules with two layers. 2L25, 25 mg/mL glutathione enriched (FUR/CHIT) capsules with two layers. 4L25 25 mg/mL glutathione enriched (FUR/CHIT)_2_ capsules with four layers. ECAPS, empty (CHIT/FUR)_2_ capsules. Results are expressed as mean ± SD (*n* = 9). Statistical significance was determined using ANOVA and Duncan’s a posteriori test *p* ≤ 0.05. Columns for proliferation tests with different letters are statistically significant (*p* ≤ 0.05). Differences after 24 h of the experiment are marked in capital letters (A, B) and differences after 48 h with lowercase letters (a, b). Values for cytotoxicity test compared to an UC * *p* ≤ 0.05.

The determination of cell proliferation with the BrdU method consists of determining the effect of the test material on DNA synthesis in cells by measuring the binding degree of BrdU. In our research, 24 h into the experiment, the application of digested and absorbed glutathione for all examined concentrations (GSH5, GSH25), glutathione enriched (FUR/CHIT)_n_ capsules (2L5, 2L25, 4L25) and empty capsules (ECAPS) did not reduce the proliferation of BJ normal cells statistically significantly (*p* > 0.05) compared to the control group. Extending the experiment for an additional 24 h led to significant inhibition (*p* ≤ 0.05) of the cell line’s proliferation (Figure 3A). The obtained results revealed that incubation with digested and absorbed capsules reduced cell growth of Hep G2 cells after 24 and 48 h (Figure 3B). The addition of GSH5 to the Hep G2 cancer cell culture led to statistically significant (*p* ≤ 0.05) inhibitions of cell proliferation of 23% and 39% after 24 h and 48 h of incubation, respectively (Figure 3B). In turn, the addition of 2L5 or 2L25 capsules into the Hep G2 cell culture resulted in reductions of cell proliferation rates by 17.60% and 15% after 24 h of incubation. Extending the experiment for an additional 24 h led to a significant (*p* ≤ 0.05) inhibition of proliferation by 37% and 44% (Figure 3B). To sum up, the highest statistically significant (*p* ≤ 0.05) decrease in the proliferation rate was observed following 48 h of incubation with 2L25.

Research has demonstrated that chitosan exhibits inhibitory effects on the growth and proliferation of diverse cancer cell lines, including Hep G2. In studies, it has been reported that edible chitosan and alginate coating treatment can reduce the proliferation of the Hep G2 human hepatocyte carcinoma cell line in a dose-dependent manner [22]. Furthermore, the investigators noted no statistically significant decline in the proliferation rate of the control normal BJ cell line, which partly corresponds to our research results. Only a limited number of scientific studies have focused on studying the effects of furcellaran on cancer cells.

### 3.3. Cytotoxicity

LDH is an enzyme that is discharged into the cell culture medium when the integrity of the cell membrane is compromised, signaling cellular damage or cell demise. The cytotoxic effects of digested and absorbed capsules on the BJ and Hep G2 cell lines were assessed following 24 h and 48 h of incubation (Figure 3C,D). Treatment with all of the examined capsules did not cause a cytotoxic effect or necrosis in normal cells after 24 h exposure. None of the tested nanocapsules exhibited cytotoxic effects or reached an IC50 value in relation to the BJ normal cell line or the Hep G2 cancer cell line. After 48 h, the highest cytotoxicity levels in the BJ cell line were observed in the samples incubated with GSH5 and 2L25. However, the number of killed cells was below 10% at all the tested time points (Figure 3C). In turn, GSH5 had a statistically significant (*p* ≤ 0.05) toxic effect on the cancer cells at the 48 h time point. Moreover, an increase in toxicity above 10% against Hep G2 cells for 2L25 was observed at all the tested time points (Figure 3D).

Chitosan is generally regarded as biocompatible and devoid of toxicity. However, its cytotoxic effects can fluctuate depending on various factors, such as concentration, molecular weight, duration of treatment, degree of deacetylation, and the specific characteristics of the cancer cell line being studied [23,24]. According to Such et al. [22] regarding the Hep G2 cancer cell line, of the tested polysaccharide edible coating complexes, no cytotoxic effects were observed, IC50 was not reached, and there was no significant decrease in cell proliferation, even at the highest concentrations tested. Another study indicated that chitosan exhibits minimal cytotoxicity on Caco-2 cells; however, effective concentrations for cell cytotoxicity were observed at higher levels (ranging from 3.2 to >20 mg/mL) [23]. It has been observed that the use of high molecular weight chitosan as a polymer-carrier for intravenous administration might lead to potential cytotoxic effects. Nonetheless, differences in chitosan’s binding affinities to cell membranes may result in differing degrees of cytotoxicity [25]. Research has confirmed that, considering safety and absorption profile considerations, chitosan oligosaccharides can be regarded as safe and promising candidates for pharmaceutical and biomedical applications.

### 3.4. PI3K/MAPK Activity Assay

To further characterize the mechanism by which examined capsules suppress the cell growth of Hep G2 cells, intracellular pathways (MAPK–Mitogen-Activated Protein Kinase and PI3K–Phosphatidylinositol 3-Kinase) known to be activated during cancer cell proliferation were analyzed. Several of these signal transduction pathways play critical roles in cellular functions such as growth, proliferation, survival, and differentiation. Although the MAPK and PI3K pathways are distinct, they can interact and cross-talk with each other, leading to complex signaling networks. The MAPK pathway, a well-conserved signaling cascade, facilitates the transmission of signals from the cell surface to the nucleus, culminating in alterations in gene expression and cellular behavior. The PI3K pathway is involved in controlling cell growth, survival, metabolism, and cytoskeletal rearrangements [26].

The Muse^®^ PI3K/MAPK Activation Dual Detection Kit was utilized to evaluate the activation status of phosphorylated AKT and ERK1/2 concurrently in the cell lines. Subsequently, analysis of the cells was conducted using a Muse^®^ Cell Analyzer. The Muse^®^ analysis software (v.1.5) was used to estimate the percentage of cells exhibiting various activation states: those negative for AKT and ERK1/2 activation (indicating no activation of the PI3K or MAPK pathways), those with ERK1/2 activation (indicating activation of the MAPK pathway), those with AKT activation (indicating activation of the PI3K pathway), and those with dual pathway activation (indicating activation of both pathways). As shown in Table 1 and Figure S1, 0.97 ± 0.46% of control cells of the MAPK pathway, 25.27 ± 1.79% exhibited activation of the PI3K pathway was activated, 64.67 ± 0.96% exhibited activation of both pathways, and 9.1 ± 0.35% of the cells showed no activation of either of them. Treatment with all of the tested capsules resulted in a significant (*p* ≤ 0.05) reduction in the percentage of cells exhibiting dual activation of ERK1/2 and AKT, with values decreasing from 43.73 ± 2.63% for the 2L5 material to 29 ± 2.9% for the ECAPS material. At the same time, the number of cells negative for ERK1/2 and AKT activation increased from 13.03 ± 1.8% for the 2L5 material to 17.33 ± 1.0% for the 2L25 material in comparison to the control (Table 1 and Figure S1). The treatment of Hep G2 cancer cells with 2L25 capsules resulted in the highest increase in the number of cells with no activation of the PI3K or MAPK pathways (17.33 ± 1.0%), with a concomitant decrease in cells with activated pathways compared to the control (30.03% ± 1.44%) (Table 1 and Figure S1). These results, suggest that the capsules under investigation effectively inhibit the MAPK/ERK and PI3K/AKT pathways in Hep G2 cancer cells.

This study showed for the first time that chitosan and furcellaran capsules enriched with glutathione, as well as glutathione itself, significantly decreased dual AKT/PI3K pathway activation while also decreasing the activation of the MAPK pathway alone in Hep G2 cells, which is a promising phenomenon. However, levels of PI3K were increased in the cancer cell line. The down-regulation of the MAPK pathway resulted from the up-regulation of PI3K, as the PI3K pathways can intersect through cross-talk among their downstream effectors [27,28].

By maintaining the cellular redox balance, glutathione can influence the activity of MAPK pathway components. Oxidative stress, which depletes intracellular glutathione, can activate MAPK signaling. Increased levels of glutathione or exogenous glutathione supplementation can help reduce oxidative stress and modulate MAPK pathway activity. Therefore, the antioxidant properties of glutathione may attenuate MAPK activation and downstream signaling events.

Glutathione may also have the potential to regulate the PI3K pathway by affecting the cellular redox state. The depletion of intracellular glutathione induced by oxidative stress can trigger PI3K signaling, resulting in heightened phosphorylation and activation of AKT. Conversely, increased levels of glutathione or exogenous glutathione supplementation can counteract oxidative stress and potentially attenuate PI3K pathway activation. The antioxidant properties of glutathione may help maintain redox balance and regulate PI3K pathway signaling.

Apoptosis is as a critical mechanism in controlling cell proliferation and eliminating damaged or abnormal cells, including cancerous ones. Many chemopreventive and chemotherapeutic agents exert their effects by promoting apoptosis in cancer cells, thereby impeding their growth. The mechanisms by which bioactive compounds induce apoptosis should be further investigated to find their mode of action and potential targets within cancer cells. Examining the potential of the tested capsules to induce apoptosis, in addition to inhibiting cell proliferation, may be an essential element in evaluating their efficacy.

### 3.5. Detection of Early and Late Markers of Biochemical Apoptosis

One of the key events that occur during the early stages of apoptosis is the externalization of phosphatidylserine (PS) to the outer surface of the cell membrane. The Muse^®^ Annexin V & Dead Cell Assay is a flow cytometry-based method that leverages the binding of annexin V to phosphatidylserine to identify and quantify apoptotic cells [29]. The Muse^®^ Caspase-3/7 Kit is designed to measure the activation of caspase-3 and caspase-7, which are initiator caspases involved in the early stages of apoptosis. Caspase-3 and caspase-7 play pivotal roles in executing the terminal events of the apoptotic pathway by cleaving various cellular substrates. A dead cell dye, 7-AAD (7-aminoactinomycin D) is used to assess cell membrane integrity and distinguish between live cells, early apoptotic cells, and dead cells. The analysis of the results obtained corroborated the capacity of the examined capsules to stimulate apoptosis within the Hep G2 cancer cell line (Table 2 and Figure S2). In the current study, we observed that digested and absorbed capsules statistically significantly (*p* ≤ 0.05) decreased the count of the live cell population compared to the UC after 48 h of incubation (Table 2 and Figure S2). The significant increase (*p* ≤ 0.05) in the total apoptosis rate further supports the notion that treatment with the examined capsules had an effect on inducing apoptosis in the treated cells (Table 2 and Figure S2). The highest number of live cells was observed for those treated with ECAPS and 4L25 (66.3± 3.29% and 67.58 ± 0.72%, respectively). Importantly, the lowest apoptotic effect was observed in the Hep G2 cell line treated with the same capsules—ECAPS and 4L25. The total apoptosis rate was 31.70 ± 3.23% and 30.75 ± 1.13, respectively (Table 2 and Figure S2). In the Hep G2 cell line, we found the highest total apoptosis rates of 38.72 ± 2.44% for 2L25 and 37.82 ± 0.38% for 2L5 biopolymer capsules (Table 2 and Figure S2). Early apoptosis was significantly increased in cells treated with GHS5, GSH25, 2L5, and 2L25 in comparison to the control medium (Table 2 and Figure S2). Late-stage biochemical apoptosis was assessed using the Muse^®^ Caspase-3/7 Kit. The caspase-3 and caspase-7 assays allowed us to identify a significant (*p* ≤ 0.05) decrease in the live cell populations in the digested and absorbed capsule treatment compared to the medium control in the Hep G2 cancer cell line (Table 2 and Figure S2). The treatment with all examined capsules led to a significant increase in the apoptotic cell population (Table 2 and Figure S2). Again, the highest number of live cells was observed in samples treated with empty capsules and 4L25 (51.44 ± 0.72% and 57.37 ± 0.86, respectively). When the cells were treated for 48 h, a more pronounced activation of apoptosis was observed for 2L5 and 2L25 biopolymer capsules compared to the control (60.68 ± 1.05 and 56.37 ± 0.8, respectively) (Table 2 and Figure S2). Furthermore, after the treatment using the capsules, the rate of late apoptosis exceeded that of the earlier treatment (Table 2 and Figure S2).

The Muse^®^ BCL-2 Activation Dual Detection Assay Kit is a valuable tool for studying the levels of activated and non-activated BCL-2 protein in cells. The BCL-2 family of proteins plays several crucial roles in regulating mitochondrial pathway of apoptosis [30]. The phosphorylation of BCL-2 protein enhances its anti-apoptotic function. Conversely, dephosphorylation of BCL-2 can lead to its inactivation or conversion into a pro-apoptotic protein, allowing for the release of pro-apoptotic factors from the mitochondria and initiation of the apoptotic cascade. All of the examined capsules induced in the Hep G2 liver cancer cell line the inactivation of the BCL-2 protein (Table 2 and Figure S2). The findings suggest that the capsules, notably the digested and absorbed GSH5, GSH25, 2L5, and 2L25 capsules, cause significant (*p* ≤ 0.05) alterations in the phosphorylation status of the BCL-2 protein within the Hep G2 liver cancer cell line. Specifically, a reduction in BCL-2 phosphorylation to about 85–86% compared to the control was observed after incubation with these capsules (Table 2 and Figure S2). The observation that the mentioned capsules were more effective at inactivating the BCL-2 protein compared to the ECAPS and 4L25 capsules is noteworthy (Table 2 and Figure S2).

Apoptosis is a highly organized form of cell death crucial for normal development and tissue homeostasis across all organisms. Despite significant knowledge about the components of apoptosis and its triggers, there are still major unanswered questions regarding the regulatory mechanisms of this multi-step process. Apoptosis can be triggered by various mechanisms, such as DNA damage, the disruption of mitochondrial function, or the activation of specific signaling pathways. Glutathione plays a pivotal role in modulating cell functions, serving as a crucial component in cellular defense against oxidants, regulating protein thiols’ redox state, and maintaining the redox homeostasis essential for proper cellular processes, including apoptosis [31]. Recent evidence has shown elevated levels of GSH in various human cancer tissues such as breast, colon, lung, bone marrow, ovarian, or larynx cancers [32]. It is well known that GSH deficiency, caused for example by drugs, sensitizes cells to anticancer treatment. For instance, glutathione depletion leads to cell growth inhibition and heightened apoptosis in pancreatic cancer cells [33]. Therefore, the question of how to lower the concentration of glutathione in cancer cells and thus increase their sensitivity to treatment arises. Research linking dietary intake of GSH with elevated blood levels and decreased cancer risk supports the use of orally administered GSH for this purpose [34]. The self-prescribed implementation of glutathione among cancer patients is a phenomenon observed in some cases, driven by the intention to reduce the toxic effects of anticancer treatments and potentially protect normal tissues from damage [35]. However, oral administration of glutathione faces several challenges. The thiol group of GSH is highly reactive and can be enzymatically and non-enzymatically oxidized to its oxidized form, glutathione disulfide (GSSG), which lacks biological activity. Additionally, GSH is susceptible to chemical and enzymatic breakdown by digestive enzymes. This hydrolysis process breaks down GSH into its constituent amino acids, resulting in a loss of biological activity. Enclosing GSH in a protective multilayer material may reduce its susceptibility to enzymatic and chemical degradation during passage through the gastrointestinal tract. This approach shows promise for increasing the bioavailability and effectiveness of orally administered glutathione supplements, particularly in the context of potential therapeutic applications [36]. GSH has also been discovered to be absorbed and transported across the human intestinal epithelial cells in vitro [37].

The research findings reported by Perego et al. [38] describe the potential mechanisms underlying the anti-proliferative effect of exogenous glutathione in A2780 ovarian carcinoma cells. They suggested that treatment with exogenous glutathione led to the generation of DNA damage within the A2780 cells, which in turn, triggered levels of apoptosis. Shen et al. [39] observed the dual role of glutathione (GSH) in selenite (Se)-induced oxidative stress and apoptosis in human HepG2 hepatoma cells [39]. Their results suggest that the intracellular level of GSH plays a critical role in modulating the response to Se-induced oxidative stress and apoptosis.

Polysaccharide-based capsules have been explored as potential carriers for delivering anticancer agents to tumor cells. Chitosan can be a safe and controlled way to deliver active substances. It has been shown in studies that chitosan-based nanoparticles or drug conjugates can enhance the cytotoxic effects of anticancer drugs on cancer cell lines, including Hep G2. Milosavljevic et al. [1] enclosed the cancer drug doxorubicin within furcellaran-chitosan coatings. Research on malignant cell lines (the MDA-MB-231 breast cancer cell line and PC3 prostate cancer cell line) has revealed the highly effective anticancer effects of the tested nanomaterial. Similar results of tests on Hep G2 cells demonstrated that curcumin nanoparticles coated with chitosan and poly(butyl)cyanoacrylate could induce better necrosis and apoptosis compared to curcumin alone [40].

In our research, 2L5 and 2L25 capsules caused the highest total apoptosis rate in cancer cells. Recently, chitosan has gained attention and use in the medical and pharmaceutical fields due to its low toxicity and various biological effects. Hasegawa et al. [41] investigated the growth-inhibitory effects of chitosan on cancer cells. Their findings reveal that chitosan treatment resulted in DNA fragmentation and increased caspase-3 activity. Glutathione was found to induce apoptosis by triggering the cleavage of poly(ADP-ribose) polymerase and generation of the pro-apoptotic protein Bax.

### 3.6. Cell Cycle Phase Distribution

To investigate whether the suppression of Hep G2 cell proliferation is associated with cell cycle arrest, we conducted a cell cycle analysis. The Muse^®^ Cell Cycle Kit identifies cells at various stages of the cell cycle by measuring DNA content. Changes in cell DNA levels are measured fluorescently using propidium iodide as a dye. In the G0/G1 phase, often referred to as the first gap phase, the cell undergoes physical enlargement, replicates organelles, and synthesizes molecular components required for subsequent stages. Before the division of the cell, new DNA is synthesized (S phase) until the chromosomal DNA is completely doubled (G2/M phase). Cells in the G2/M phase eventually divide into two cells, and the cell cycle either starts anew or is stopped. In the cancer cell line under examination, the tested capsules induced notable alterations in cell cycle distribution compared to UC. As shown in Table 3 and Figure S3, treatment of Hep G2 cancer cells with all the examined capsules resulted in a significantly (*p* ≤ 0.05) higher percentage of cells in the G2/M phase compared to the control group, accompanied by a decrease in the percentage of cells in the S phase. A more pronounced arrest of the G2/M phase was observed for GHS5 (34.33 ± 1.38%) (Table 3 and Figure S3).

Given the observed ability of the studied capsules to induce cell death, we investigated their effects on cell cycle perturbations. The significant decrease in cell proliferation and the statistical increase in the sub-G2/M fraction of the cell cycle during capsule treatments suggests increased cell death in comparison to the controls after 48 h of treatment. As previously mentioned, proliferation was assessed using the BrdU assay, which measures the incorporation of the thymidine analogue 5-bromo-2′-deoxyuridine into DNA during synthesis, specifically during the S phase of the cell cycle. In Hep G2 cells treated with capsules, the largest population of cells was found in the G2/M phase. Therefore, differences in the BrdU assay were detectable because cell arrest occurred in either this phase or the subsequent phase of the cell cycle. Similar to our study, previous research has indicated a noticeable G2/M block when cancer cells were exposed to varying doses of extracellular GSH [38]. According to Salehi et al. [42], chitosan, as a polysaccharide with notable biological and anticancer properties, exerts an inhibitory effect on the proliferation of breast cancer cells, as well as cell cycle arrest. In another study, it was shown that chitosan treatment causes cell cycle arrest in several types of cancer, including oral cancer. It can inhibit cell cycle progression, leading to a halt in cell division and reduced cell viability. Chitosan-induced cell cycle arrest has been observed in different phases of the cell cycle, depending on the specific experimental conditions [43]. The findings of this study agree with those from previous studies that indicate that chitosan nanoparticles induced G2/M phase cell cycle arrest in Hep G2 cells, leading to reduced cell viability [44].

## 4. Conclusions

Two- and four-layer FUR/CHIT-based microcapsules with GSH were successfully produced. In addition, an empty FUR/CHIT-based capsule was effectively prepared, which may be a potential model carrier for sensitive active ingredients. An important observation of our study is the connection between the inhibition of proliferation and the ability of GSH (alone or in FUR/CHIT layers) to induce apoptosis in cancer cell lines. Taken together, our findings suggest that the tested capsules, in particular 2L5 and 2L25, induced cell death through an apoptotic pathway. The presented data also allow us to suggest that the inhibition of cell proliferation or induction of cell death in Hep G2 cancer cells by the examined capsules is primarily linked to the induction of G2/M cell cycle arrest. It is important to note that their effectiveness and clinical applications must be investigated. Further research is needed to evaluate their safety, pharmacokinetics, and therapeutic efficacy in preclinical and clinical trials. Additional studies are necessary to assess their cytotoxicity across different cell types and to evaluate their biocompatibility in relevant animal models.

## Figures and Tables

**Table 1:** MAPK and PI3K pathway activation in Hep G2 cancer cells treated with experimental capsules.

PI3K/MAPK Activation								
	UC	STS	GSH5	GSH25	2L5	2L25	4L25	ECAPS
**MAPK activation**	0.97 ^c^ ± 0.46	0.05 ^a^ ± 0.07	0.73 ^b,c^ ± 0.25	0.83 ^c^ ± 0.25	0.83 ^c^ ± 1.21	0.3 ^a,c^ ± 0.35	0.2 ^a^ ± 0.2	0.5 ^a,b,c^ ± 0.17
**PI3K activation**	25.27 ^a^ ± 1.79	56.85 ^d^ ± 0.64	45.0 ^b^ ± 1.14	42.87 ^b^ ± 1.1	42.4 ^b^ ± 0.96	52.07 ^c^ ± 1.99	54.4 ^c,d^ ± 1.47	53.9 ^c^ ± 2.14
**dual pathway activation**	64.67 ^e^ ± 0.96	16.17 ^a^ ± 0.57	39.63 ^c^ ± 2.0	40.07 ^c^ ± 2.39	43.73 ^d^ ± 2.63	30.3 ^b^ ± 1.44	29.0 ^b^ ± 2.8	29.13 ^b^ ± 0.46
**negative**	9.1 ^a^ ± 0.35	26.4 ^d^ ± 1.13	14.63 ^b,c^ ± 1.12	16.23 ^b,c^ ± 3.0	13.03 ^b^ ± 1.8	17.33 ^c^ ± 1.0	16.4 ^c^ ± 1.81	16.47 ^c^ ± 2.65

UC, untreated control. GSH5, 5 mg/mL glutathione. GSH25, 25 mg/mL glutathione. 2L5, 5 mg/mL glutathione enriched FUR/CHIT capsules with two layers. 2L25, 25 mg/mL glutathione enriched (FUR/CHIT) capsules with two layers. 4L25 25 mg/mL glutathione enriched (FUR/CHIT)_2_ capsules with four layers. ECAPS, empty (CHIT/FUR)_2_ capsules. The results are presented as mean ± standard deviation (*n* = 3). Differences that are statistically significant (determined by ANOVA and Duncan’s post hoc test) with *p* ≤ 0.05 within each row are denoted by letters (a–e).

**Table 2:** Results of cell apoptosis in Hep G2 following treatment with experimental capsules.

**Apoptosis Activity**								
	**UC**	**STS**	**GSH5**	**GSH25**	**2L5**	**2L25**	**4L25**	**ECAPS**
**Live**	85.98 ^e^ ± 0.88	55.93 ^a^ ± 0.93	62.67 ^c^ ± 1.62	62.65 ^c^ ± 1.4	59.15 ^b^ ± 0.13	60.13 ^b,c^ ± 1.92	66.3 ^d^ ± 3.29	67.58 ^d^ ± 0.72
**Early apoptotic**	7.78 ^a^ ± 0.43	22.78 ^c^ ± 1.58	16.98 ^b^ ± 1.19	14.52 ^b^ ± 1.58	17.95 ^b^ ± 0.75	15.10 ^b^ ± 3.88	9.9 ^a^ ± 2.08	10.0 ^a^ ± 1.16
**Late apoptotic**	3.40 ^a^ ± 2.0	19.55 ^b,c^ ± 0.2	17.60 ^b^ ± 0.78	19.47 ^b,c^ ± 0.76	19.87 ^b,c^ ± 0.43	23.62 ^d^ ± 1.73	21.80 ^c,d^ ± 1.73	20.75 ^c^ ± 1.57
**Dead**	1.15 ^a^ ± 0.0	1.4 ^a,b^ ± 0.1	2.75 ^c^ ± 0.41	3.37 ^d^ ± 0.4	3.03 ^c,d^ ± 0.33	1.15 ^a^ ± 0.52	2.0 ^b^ ± 0.1	1.67 ^a,b^ ± 0.43
**Total apoptotic**	12.88 ^a^ ± 0.88	42.68 ^e^ ± 1.03	34.58 ^c^ ± 1.85	37.82 ^b,c^ ± 0.38	37.82 ^d^ ± 0.38	38.72 ^d^ ± 2.44	31.70 ^b,c^ ± 3.23	30.75 ^b^ ± 1.13
**Caspase-3/7 Activity**								
	**UC**	**STS**	**GSH5**	**GSH25**	**2L5**	**2L25**	**4L25**	**ECAPS**
**Live**	89.8 ^e^ ± 0.35	46.47 ^b,c^ ± 2.8	43.17 ^b^ ± 9.56	48.28 ^b,c^ ± 1.51	36.50 ^a^ ± 1.26	41.78 ^a,b^ ± 1.08	51.44 ^c,d^ ± 0.72	57.37 ^d^ ± 0.86
**Early apoptotic**	2.93 ^a^ ± 0.53	31.87 ^e^ ± 4.59	19.58 ^c,d^ ± 5.25	7.18 ^a^ ± 0.35	21.15 ^d^ ± 1.26	16.32 ^b,c^ ± 0.85	17.16 ^b,c,d^ ± 0.96	14.27 ^b^ ± 0.15
**Late apoptotic**	7.13 ^a^ ± 0.68	21.55 ^b^ ± 2.07	30.47 ^c^ ± 7.57	39.43 ^d^ ± 2.32	39.83 ^d^ ± 1.89	40.05 ^d^ ± 1.03	29.31 ^c^ ± 0.7	26.43 ^b,c^ ± 1.05
**Dead**	0.13 ^a^ ± 0.14	0.12 ^a^ ± 0.08	6.78 ^b^ ± 7.73	4.43 ^a,b^ ± 0.51	2.52 ^a,b^ ± 0.21	1.85 ^a,b^ ± 0.31	2.09 ^a,b^ ± 0.14	1.99 ^a,b^ ± 0.15
**Total apoptotic**	10.07 ^a^ ± 0.3	53.45 ^d,e^ ± 2.70	50.05 ^c,d^ ± 6.08	46.62 ^c^ ± 2.16	60.68 ^f^ ± 1.05	56.37 ^e^ ± 0.8	46.47 ^c^ ± 0.64	40.69 ^b^ ± 0.99
**BCL-2 Activation**								
	**UC**	**STS**	**GSH5**	**GSH25**	**2L5**	**2L25**	**4L25**	**ECAPS**
**Activated**	82.6 ^d^ ± 1.51	7.53 ^a^ ± 0.84	14.03 ^a,b^ ± 1.89	12.9 ^a,b^ ± 1.68	15.3 ^b^ ± 2.19	15.47 ^b^ ± 1.81	26.8 ^c^ ± 9.07	24.72 ^c^ ± 3.51
**Inactivated**	17.3 ^a^ ± 1.51	91.87 ^d^ ± 0.67	85.2 ^c,d^ ± 1.65	86.2 ^c,d^ ± 1.9	83.5 ^c^ ± 2.29	83.5 ^c^ ± 1.61	72.23 ^b^ ± 9.26	74.92 ^b^ ± 3.42
**Non-expressing**	0.1 ^a^ ± 0.0	0.53 ^a,b,c^ ± 0.21	0.67 ^b,c,d^ ± 0.38	0.83 ^c,d^ ±0.31	1.13 ^d^ ± 0.5	0.93 ^c,d^ ± 0.31	0.63 ^a,b,c,d^ ± 0.15	0.21 ^a,b^ ± 0.1

UC, untreated control. GSH5, 5 mg/mL glutathione. GSH25, 25 mg/mL glutathione. 2L5, 5 mg/mL glutathione enriched FUR/CHIT capsules with two layers. 2L25, 25 mg/mL glutathione enriched (FUR/CHIT) capsules with two layers. 4L25 25 mg/mL glutathione enriched (FUR/CHIT)_2_ capsules with four layers. ECAPS, empty (CHIT/FUR)_2_ capsules. The results are presented as mean ± standard deviation (*n* = 3). Statistically significant differences (determined by ANOVA and Duncan’s post hoc test) with *p* ≤ 0.05 within each row are denoted by letters (a–f).

**Table 3:** Change regarding cell cycle phases of Hep G2 line cancer cells treated with experimental material.

Cell Cycle Phase Distribution								
	UC	STS	GSH5	GSH25	2L5	2L25	4L25	ECAPS
**G0/G1**	41.80 ^c^ ± 0.79	26.7 ^a^ ± 2.19	42.47 ^c^ ± 3.26	36.67 ^b^ ± 1.72	37.3 ^b^ ± 1.68	42.17 ^c^ ± 1.95	39.37 ^b,c^ ± 2.55	37.57 ^b^ ± 2.22
**S**	29.33 ^c^ ± 0.12	24.33 ^b^ ± 1.46	21.27 ^a^ ± 1.0	22.1 ^a,b^ ± 0.87	22.77 ^a,b^ ± 0.72	21.6 ^a^ ± 0.0	24.33 ^b^ ± 2.15	23.70 ^a,b^ ± 2.36
**G2/M**	15.73 ^a^ ± 0.86	29.03 ^b^ ± 0.32	30.6 ^b,c^ ± 1.76	34.33 ^e^ ± 1.38	32.8 ^d,e^ ± 0.95	30.43 ^b,c^ ± 1.43	30.67 ^b,c^ ± 0.93	31.9 ^c,d^ ± 0.56

UC, untreated control. STS, staurosporine. GSH5, 5 mg/mL glutathione. GSH25, 25 mg/mL glutathione. 2L5, 5 mg/mL glutathione enriched FUR/CHIT capsules with two layers. 2L25, 25 mg/mL glutathione enriched (FUR/CHIT) capsules with two layers. 4L25, 25 mg/mL glutathione enriched (FUR/CHIT)_2_ capsules with four layers. ECAPS, empty capsules. The results are presented as mean ± standard deviation (*n* = 3). Statistically significant differences (determined by ANOVA and Duncan’s post hoc test) with *p* ≤ 0.05 within each row are denoted by letters (a–e).

## Data Availability

Data will be made available on request.

## Supplementary Materials

The following supporting information can be downloaded at: https://www.mdpi.com/article/10.3390/ma17092047/s1, Figure S1: MAPK and PI3K pathway activation in Hep G2 cancer cells at treated with experimental capsules; Figure S2: Cell apoptosis results of Hep G2 cancer cells at treated with experimental capsules; Figure S3: The change of cell cycle phases of cancer cells of the Hep G2 line at treated with experimental capsules.
