# Supplementary material for: Design and In Vitro Activity of Furcellaran/Chitosan Multilayer Microcapsules for the Delivery of Glutathione and Empty Model Multilayer Microcapsules Based on Polysaccharides
Source: Materials (Basel). 2024 Apr 26;17(9):2047. doi: 10.3390/ma17092047 (PMC11084246; doi:10.3390/ma17092047)
Supplement: Supplementary file 1 [file materials-17-02047-s001.zip › materials-2918136-supplementary.pdf]

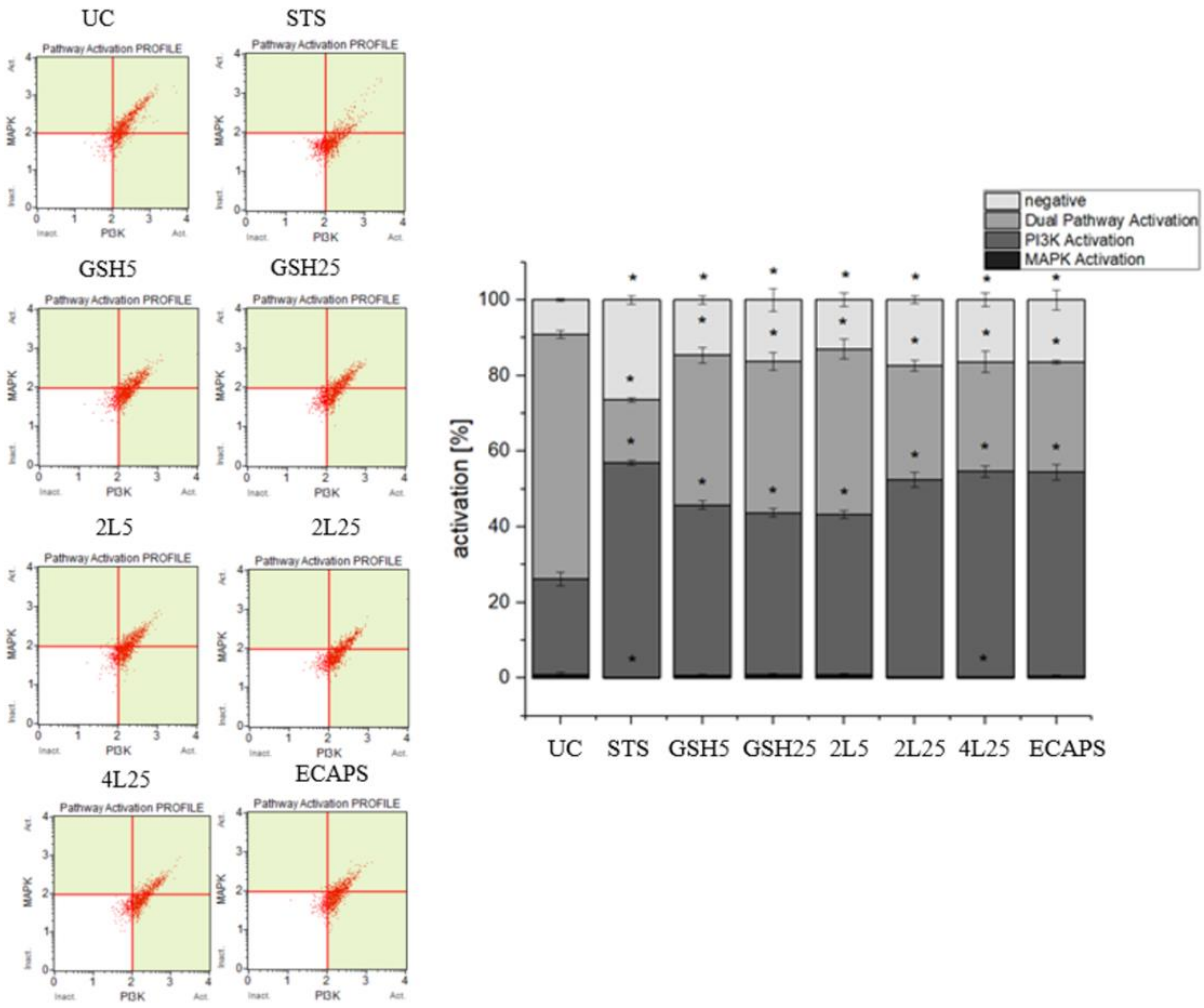


**Figure S1**. MAPK and PI3K pathway activation in Hep G2 cancer cells at treated with experimental capsules. The cells were analyzed using a Muse® Cell Analyzer. The Muse® PI3K/MAPK Activation Dual Detection Kit was used to evaluate simultaneously the activation of both phosphorylated AKT and ERK1/2 in the cell line. UC, untreated control. GSH5, 5 mg/ml glutathione. GSH25, 25 mg/ml glutathione. 2L5, 5 mg/mL glutathione-enriched FUR/CHIT capsules with 2 layers. 2L25, 25 mg/mL glutathione-enriched (FUR/CHIT) capsules with 2 layers. 4L25, 25 mg/mL glutathione-enriched $(FUR/CHIT)_2$ capsules with 4 layers. ECAPS, empty $(CHIT/FUR)_2$ capsules. The results are expressed as mean ± SD ($n = 3$). Statistical significance was based on ANOVA and Duncan's a posteriori test. * $p \leq 0.05$ compared to untreated control.

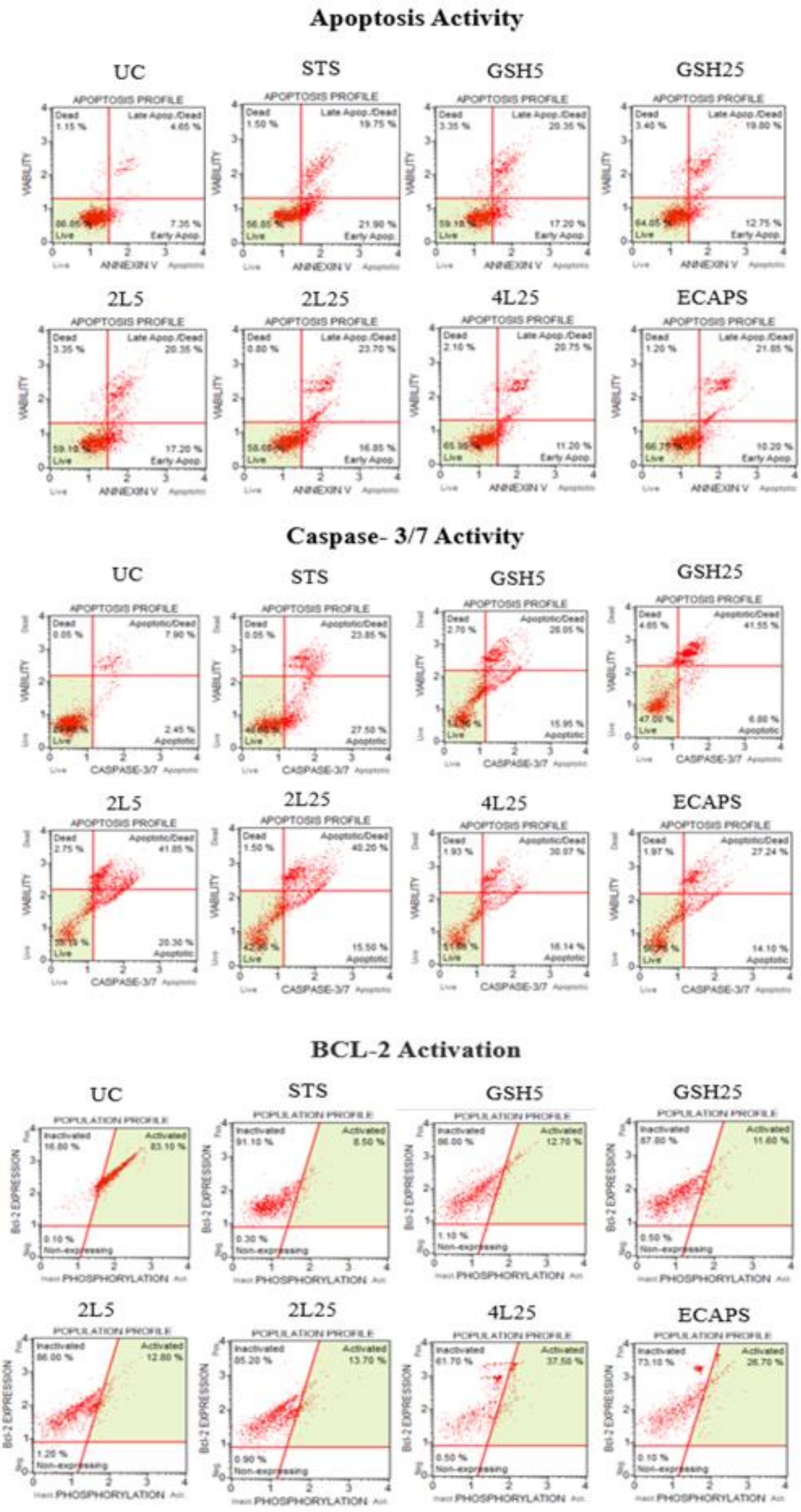
Apoptosis Activity
UC
STS
GSH5
GSH25
2L5
2L25
4L25
ECAPS
APOPTOSIS PROFILE
VIABILITY
ANNEXIN V
Caspase- 3/7 Activity
UC
STS
GSH5
GSH25
2L5
2L25
4L25
ECAPS
APOPTOSIS PROFILE
VIABILITY
CASPASE-3/7
BCL-2 Activation
UC
STS
GSH5
GSH25
2L5
2L25
4L25
ECAPS
POPULATION PROFILE
Bcl-2 EXPRESSION
PHOSPHORYLATION

**Figure S2.** Cell apoptosis results of Hep G2 cancer cells at treated with experimental capsules. The apoptosis profiling and apoptotic cell counts were obtained using flow cytometry (Muse® Cell Analyzer) and Muse® Annexin V and Dead Cell Assay Kit and Muse® Caspase- 3/7 Kit. Comparison of BCL-2 protein inactivation in cancer cells was obtained using Muse® BCL-2 Activation Dual Detection Assay Kit. UC, untreated control. GSH5, 5 mg/mL glutathione. GSH25, 25 mg/mL glutathione. 2L5, 5 mg/mL glutathione-enriched FUR/CHIT capsules with 2 layers. 2L25, 25 mg/mL glutathione-enriched (FUR/CHIT) capsules with 2 layers. 4L25, 25 mg/mL glutathione-enriched $(FUR/CHIT)_2$ capsules with 4 layers. ECAPS, empty $(CHIT/FUR)_2$ capsules.

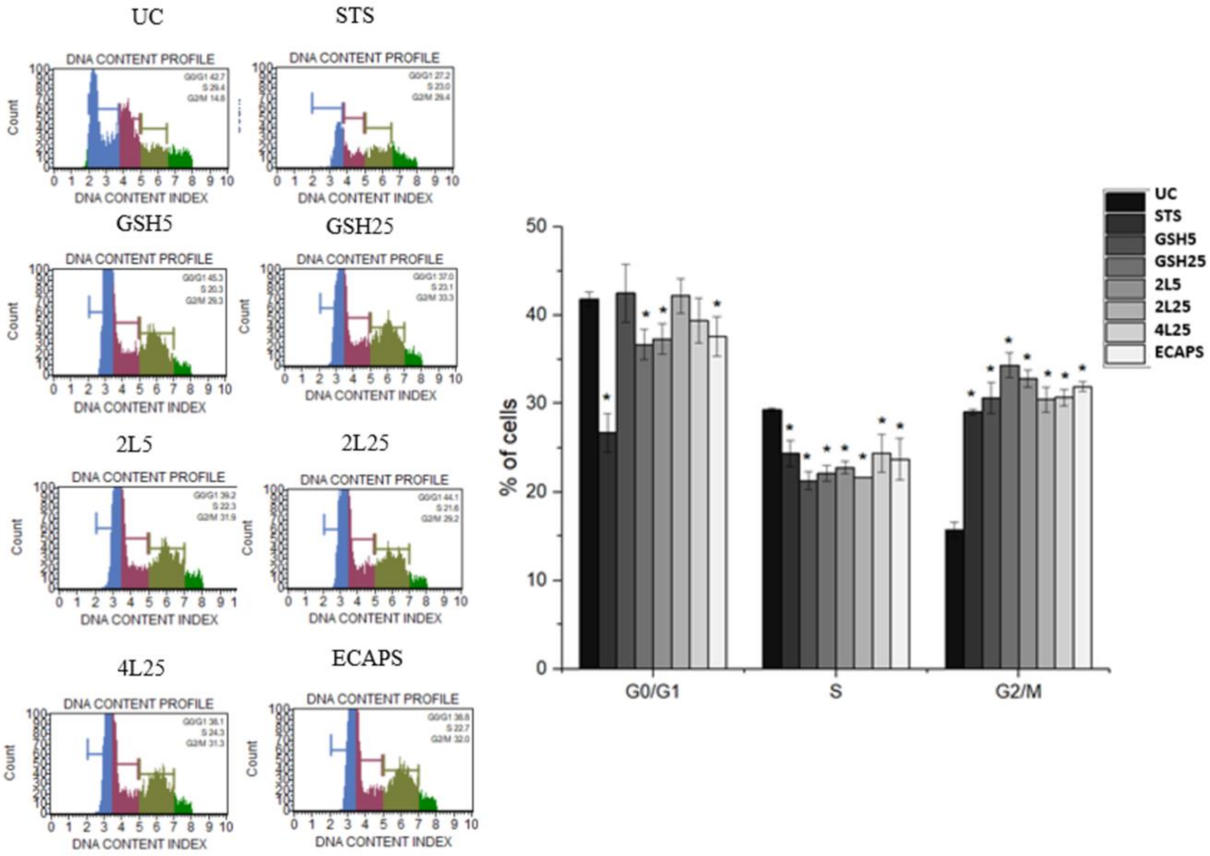


**Figure S3**. The change of cell cycle phases of cancer cells of the Hep G2 line at treated with experimental capsules. The cell cycle was analyzed using flow cytometry (Muse® Cell Analyzer) and Muse® Cell Cycle Kit. UC, untreated control. GSH5, 5 mg/mL glutathione. GSH25, 25 mg/mL glutathione. 2L5, 5 mg/mL glutathione-enriched FUR/CHIT capsules with 2 layers. 2L25, 25 mg/mL glutathione-enriched (FUR/CHIT) capsules with 2 layers. 4L25, 25 mg/mL glutathione-enriched $(FUR/CHIT)_2$ capsules with 4 layers. ECAPS, empty $(CHIT/FUR)_2$ capsules. The results are expressed as mean ± SD ($n = 3$). Statistical significance was based on ANOVA and Duncan's a posteriori test. * $p \leq 0.05$ compared to untreated control.
